# DHA-PC and PSD-95 decrease after loss of synaptophysin and before neuronal loss in patients with Alzheimer's disease

**DOI:** 10.1038/srep07130

**Published:** 2014-11-20

**Authors:** Dai Yuki, Yuki Sugiura, Nobuhiro Zaima, Hiroyasu Akatsu, Shiro Takei, Ikuko Yao, Masato Maesako, Ayae Kinoshita, Takayuki Yamamoto, Ryo Kon, Keikichi Sugiyama, Mitsutoshi Setou

**Affiliations:** 1 Department of Cell Biology and Anatomy, Hamamatsu University School of Medicine, 1-20-1 Handayama, Higashi-ku, Hamamatsu, Shizuoka 431-3192, Japan; 2 Research and Development Headquarters, Lion Corporation, 7-2-1 Hirai, Edogawa-ku, Tokyo 132-0035, Japan; 3 JST Precursory Research for Embryonic Science Technology (PREST) Project, 160-8582 Tokyo, Japan; 4 Department of Applied Biological Chemistry, Kinki University, 3327-204 Naka-machi, Nara 631-8505, Japan; 5 Choju Medical Institute, Fukushimura Hospital, 19-14 Yamanaka, Noyori-cho, Toyohashi, Aichi 441-8124, Japan; 6 Department of Medicine for Aging in Place and Community-Based Medical Education, Nagoya City University Graduate School of Medical Sciences, Nagoya, Aichi 467-8601, Japan; 7 Department of Optical Imaging, Medical Photonics Research Center, Hamamatsu University School of Medicine, 1-20-1 Handayama, Higashi-ku, Hamamatsu, Shizuoka 431-3192, Japan; 8 JST, ERATO, Sato project, Tokyo 160-8582, Japan; 9 School of Human Health Sciences, Kyoto University Graduate School of Medicine, 53 Shogoin kawahara-cho, Sakyo-ku, Kyoto 606-8507, Japan; 10 Ritsumeikan Global Innovation Research Organization, Ritsumeikan University, 1-1-1 Nojihigashi, Kusatsu, Shiga 525-8577, Japan

## Abstract

Alzheimer's disease (AD) is a progressive neurodegenerative disease that is characterized by senile plaques, neurofibrillary tangles, synaptic disruption, and neuronal loss. Several studies have demonstrated decreases of docosahexaenoic acid-containing phosphatidylcholines (DHA-PCs) in the AD brain. In this study, we used matrix-assisted laser desorption/ionization imaging mass spectrometry in postmortem AD brain to show that PC molecular species containing stearate and DHA, namely PC(18:0/22:6), was selectively depleted in the gray matter of patients with AD. Moreover, in the brain regions with marked amyloid β (Aβ) deposition, the magnitude of the PC(18:0/22:6) reduction significantly correlated with disease duration. Furthermore, at the molecular level, this depletion was associated with reduced levels of the postsynaptic protein PSD-95 but not the presynaptic protein synaptophysin. Interestingly, this reduction in PC(18:0/22:6) levels did not correlate with the degrees of Aβ deposition and neuronal loss in AD. The analysis of the correlations of key factors and disease duration showed that their effects on the disease time course were arranged in order as Aβ deposition, presynaptic disruption, postsynaptic disruption coupled with PC(18:0/22:6) reduction, and neuronal loss.

Alzheimer's disease (AD) is a progressive neurodegenerative disorder and the major cause of dementia in the elderly^1^. The main pathological hallmarks of AD are amyloid β (Aβ) plaques and hyperphosphorylated tau-containing neurofibrillary tangles^2^,^3^,^4^. Aβ is the leading candidate for the cause of neuronal loss and synaptic disruption, which causes the dementia in AD^5^,^6^,^7^

In the study of the postmortem brains of patients with AD, several researchers have reported that phosphatidylcholines (PCs) are decreased in patients with AD^8^,^9^. PCs, which are major lipid components in brain, can be subdivided into distinct molecular species depending on their composition of two fatty acids. In an analytical report of the molecular species of PCs, docosahexaenoic acid (DHA)-containing PCs (DHA-PCs) were significantly decreased in the brains of patients with AD^10^. In the central nervous system, DHA-PCs regulate the functioning of synaptic membrane-associated proteins because they affect membrane fluidity and protein-protein interactions^11^,^12^. DHA-PCs are also digested by Phospholipase A2 to produce free DHA and LysoPCs^13^. The oxidative products of free DHA, such as neuroprotectins, act as anti-apoptotic factors of neuronal cells^14^. Therefore, the decreases in DHA-PCs may be involved in the synaptic disruption and neuronal loss that occurs in AD.

The neuronal loss and synaptic disruption in AD are observed near Aβ deposition^15^,^16^, and they have been reported to closely reflect the progression of the cognitive deficits in AD^17^,^18^,^19^. The neuronal loss in AD is most prominent in the temporal and frontal cortices^20^, and the decreases in the levels of the presynaptic protein synaptophysin and the postsynaptic protein PSD-95, which reflect the synaptic disruption, are observed in the temporal and frontal cortices and the hippocampus^21^,^22^,^23^. The anatomical distribution of these substrates is important information in the study of neurodegeneration in patients with AD.

Here, we analyzed the distribution of DHA-PCs in the brain with Imaging Mass Spectrometry (IMS). IMS permits the direct analysis of biomolecules and the simultaneous visualization of the distribution of these molecules across a tissue section^24^,^25^,^26^. Matrix-Assisted Laser Desorption/Ionization (MALDI)-IMS, in particular, is practical for analytical lipid studies, and this method has revealed the distribution of PC species in mouse and human brain tissues^27^,^28^,^29^. With this technique, we analyzed the distributional changes of DHA-PCs in human brains with AD and in AD model mice and examined the association between DHA-PCs and aspects of neuronal loss and the decreases in synaptic proteins.

## Results

### The characterization of PC molecular species in the human brain

**Figure 1:**
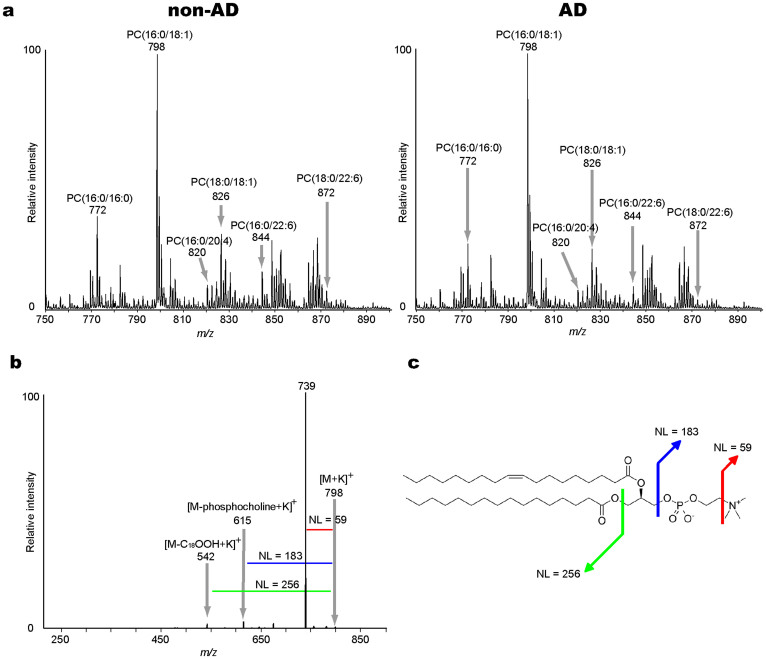
Characterization of PC molecular species in the human brain by Matrix-Assisted Laser Desorption/Ionization-Tandem Mass Spectrometry (MALDI-MS/MS). (a) The averaged mass spectra from *m/z* 750 to 900 in non-Alzheimer's Disease (AD; left panel) and AD brains (right panel). The annotations indicate peak assignments to the phosphatidylcholine (PC) molecular species with different fatty acid compositions. (b) The tandem mass spectrum of PC(16:0/18:1) at *m/z* 798 as an example of the molecular characterization by this technique. The product ions at *m/z* 739 and *m/z* 615 (from loss of trimethylamine [NL 59] and phosphocholine [NL 183] residues, respectively), were commonly observed ions formed from the PC species. The product ion at *m/z* 542 was assigned to a fragment that was formed by the neutral loss of palmitic acid (16:0). (c) The panel shows the structural formula for PC(16:0/18:1) and the assignment of the cleavage positions. NL, neutral loss.

First, we characterized the PC molecular species in the human brain with MALDI-IMS (Fig. 1). For this purpose, we performed a structural analysis with tandem mass spectrometry (MS/MS) directly on the coronal brain tissue sections of patients with and without AD. As a result, we identified six mass peaks for PCs with distinct fatty-acid compositions in both AD and non-AD specimens.

### The depletion of DHA-PC molecular species in the human temporal gray matter in MALDI-IMS

**Figure 2:**
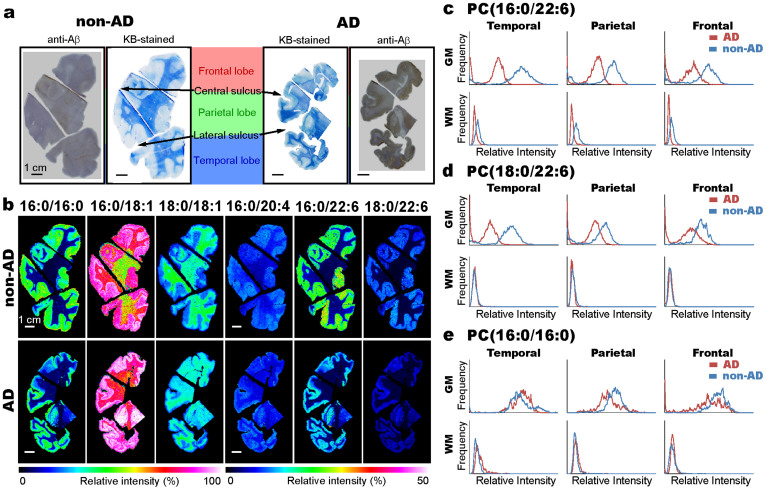
Marked reduction of docosahexaenoic acid (DHA)-PC molecular species in the temporal gray matter in AD. (a) The pictures show Aβ-immunostained and Kluver-Barrera (KB)-stained coronal sections of non-AD and AD postmortem brains. (b) The distributions of the PC species in coronal sections of non-AD and AD brains that were analyzed by MALDI-imaging mass spectrometry (IMS) with 500-µm raster step sizes. The PCs with identical fatty acid moieties were arranged horizontally. The scale bars show 1 cm. (c–e) The graphs show the histograms of the intensity distributions of PC(16:0/22:6) (c), PC(18:0/22:6) (d), and PC(16:0/16:0) (e), in different brain regions in AD and non-AD brains from the relative intensity values of the MS images.

Next, we prepared coronal brain sections, including those from the frontal, parietal, and temporal lobes, for the imaging of the characterized PCs (Fig. 2). Fig. 2a shows Kluver-Barrera (KB)-stained sections and Aβ-immunostained sections. In the AD brain, high levels of Aβ deposition were observed in the gray matter. With continuous sections, we visualized the distribution of six PC molecular species with MALD-IMS (Fig. 2b). These images show that each PC has a distinct ion intensity difference between the gray and white matter regions. Although the distribution patterns of the PCs in the AD brain were similar to those in the non-AD brain, the ion intensities of the DHA-PC molecular species, such as PC(16:0/22:6) and PC(18:0/22:6), in the AD brain were lower than those in the non-AD brain. We then performed a more detailed analysis with histograms of the intensity distributions in different brain regions (Fig. 2c–e). In gray matter regions, the intensity distribution peaks of PC(16:0/22:6) and PC(18:0/22:6) shifted to lower values in the AD brain. In addition, the shifts were clearer in the temporal lobe. In white matter regions, however, the distribution peak of PC(16:0/22:6) slightly shifted lower in the AD brain, but PC(18:0/22:6) had similar distributions between the AD and non-AD brain.

### PC(18:0/226) was markedly decreased in the gray matter in the human AD brains compared to other PC molecular species

**Figure 3:**
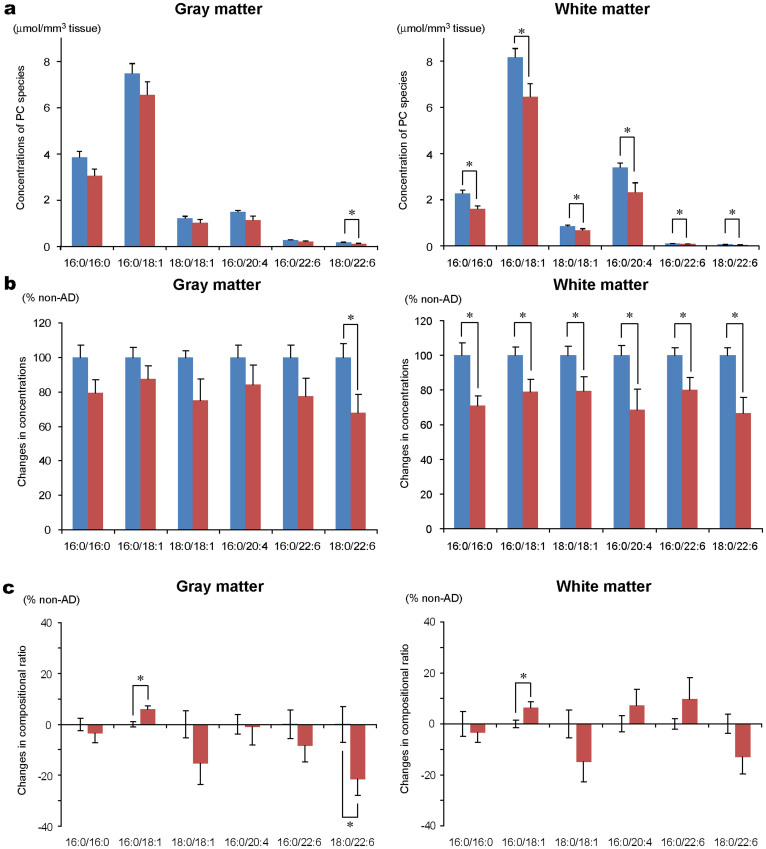
Quantitative analyses of the PC species in non-AD and AD temporal lobe with liquid chromatography-electrospray ionization (LC-ESI) MS/MS. (a) The graphs show the concentrations of the PC species in the temporal gray (left panel) and white matter (right panel) of non-AD (blue bar) and AD (red bar) brains. (b) The graphs show the % changes of the PC concentrations between non-AD (blue bar) and AD (red bar) brains in the gray (left panel) and white matter (right panel). (c) The graphs show the % change of the PC composition ratio between the non-AD and AD brains in the gray (left graph) and white matter regions (right graph). The data are shown as mean [standard error (SE)]. n = 9, * *P* < 0.05.

With MALDI-IMS, we found that the ion intensities of DHA-PCs were decreased in the gray matter of the AD brains. To examine the repeatability and specificity of this result, we performed a liquid chromatography-electrospray ionization (LC-ESI) MS/MS analysis as an alternative quantitative method (Fig. 3) in the temporal lobes of nine non-AD and nine AD patients (Table 1). Fig. 3a shows the concentrations of all of the PCs in the gray matter and white matter regions of the non-AD and AD brains. Fig. 3b shows the changes in the concentrations of the PCs with the AD values as percentages of the non-AD values. All of the PCs were decreased both in the gray and white matter in the AD brains. In particular, PC(18:0/22:6) was decreased significantly in the gray matter. When investigating the compositional ratios of the PC species, we found that the ratio of PC(18:0/22:6) was clearly and specifically decreased in the gray matter (Fig. 3c). Therefore, both of the MALDI-IMS and LC-ESI MS/MS analyses showed a marked depletion of PC(18:0/22:6) in the temporal gray matter of the AD brains.

### The compositional ratio of PC(18:0/22:6) correlated with disease duration

**Figure 4:**
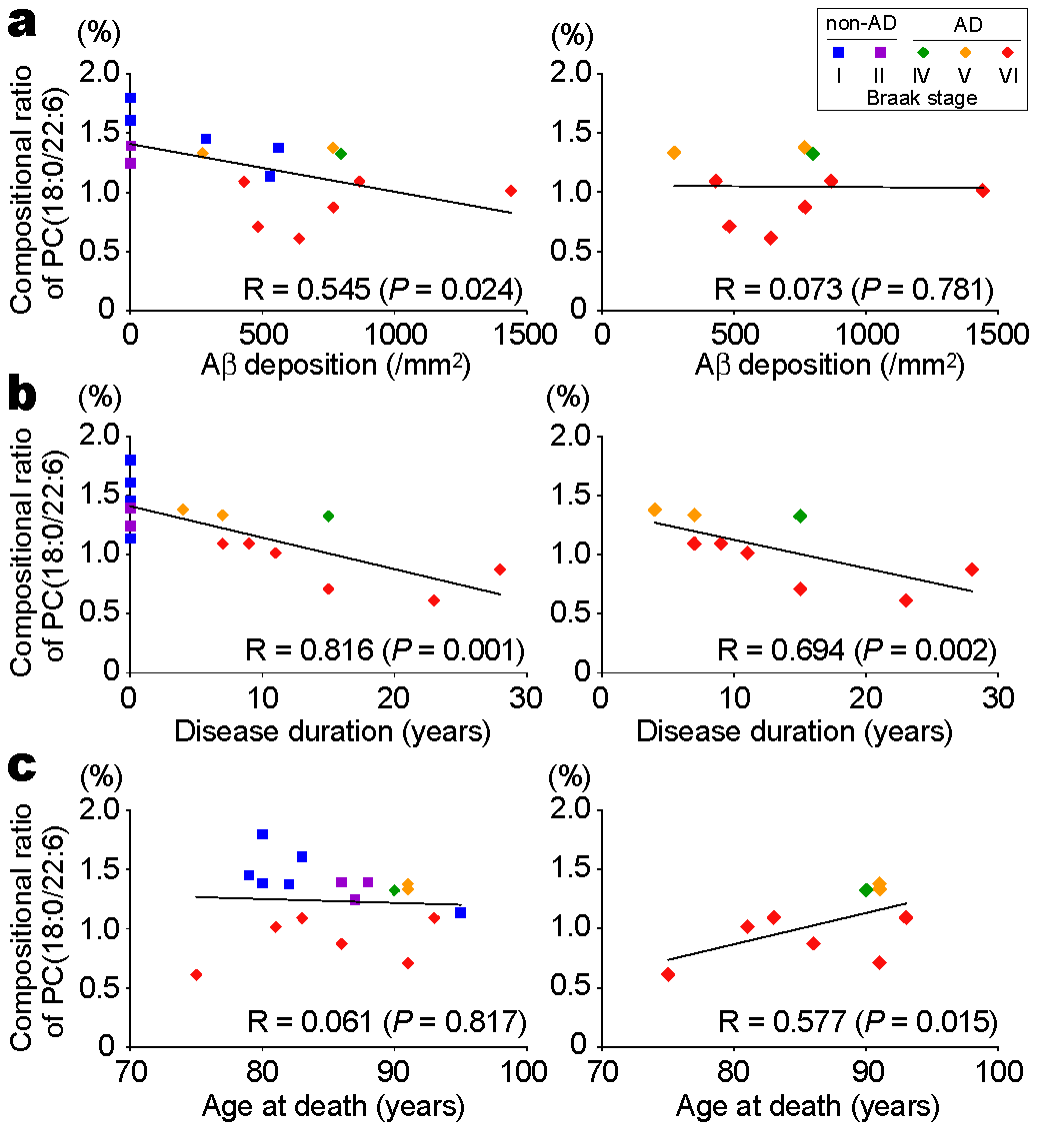
Correlations between the compositional ratio of PC(18:0/22:6) in the gray matter versus Aβ deposition, disease duration of AD, and age at death. (a) The compositional ratio of PC(18:0/22:6) in the gray matter plotted against Aβ deposition in non-AD and AD patients. (b) The compositional ratio of PC(18:0/22:6) in the gray matter plotted against the disease duration of AD in non-AD and AD patients. (c) The compositional ratio of PC(18:0/22:6) in the gray matter plotted against age at death in non-AD and AD patients. The left panels show analyses of all of the patients (non-AD and AD). The right panels show analyses of the AD patients only. A Pearson's test was used to determine the correlations between parameters.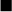 = non-AD (n = 9); 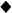 = AD (n = 9). The colors of the markers indicate the Braak stages of each subject as shown in the top-right box.

To further investigate the relationship of PC(18:0/22:6) to AD, we performed correlative analyses between the compositional ratio of PC(18:0/22:6) and Aβ density in the gray matter. Both factors correlated negatively in the analysis of the all-subjects group (R = 0.545, *P* = 0.024) but not in the analysis of the AD patients group (R = 0.073, *P* = 0.781) (Fig. 4a). We then examined the correlation with disease duration and found it to be negatively correlated with the compositional ratio of PC(18:0/22:6), both in the analysis of the all-subjects group (R = 0.816, *P* = 0.001) and in the analysis of the AD patients group (R = 0.694, *P* = 0.002) (Fig. 4b). However, the compositional ratio of PC(18:0/22:6) was not significantly correlated with the age at death in the all-subjects group (R = 0.061, *P* = 0.817) but was positively correlated with the age at death in the AD patients group (R = 0.577, *P* = 0.015) (Fig. 4c). The compositional ratio of PC(18:0/22:6) in proportion to disease duration was independent of the age of the subjects. In addition, the decrease in PC(18:0/22:6) was remarkable in patients with Braak Stage VI.

In this study, two cases of early-onset AD who had symptom onset before age 65 were included (Nos. 19 and 20 in Table 1). Because early-onset patients generally show a more rapid clinical decline than late-onset patients^30^, we also performed the correlation analyses in only the late-onset AD patients (n = 7). Even in the late-onset AD patients, the compositional ratio of PC(18:0/22:6) correlated with disease duration (R = 0.536, p = 0.027).

### The compositional ratio of PC(18:0/22:6) correlated with PSD-95 expression in the AD brains but not with neuron density

**Figure 5:**
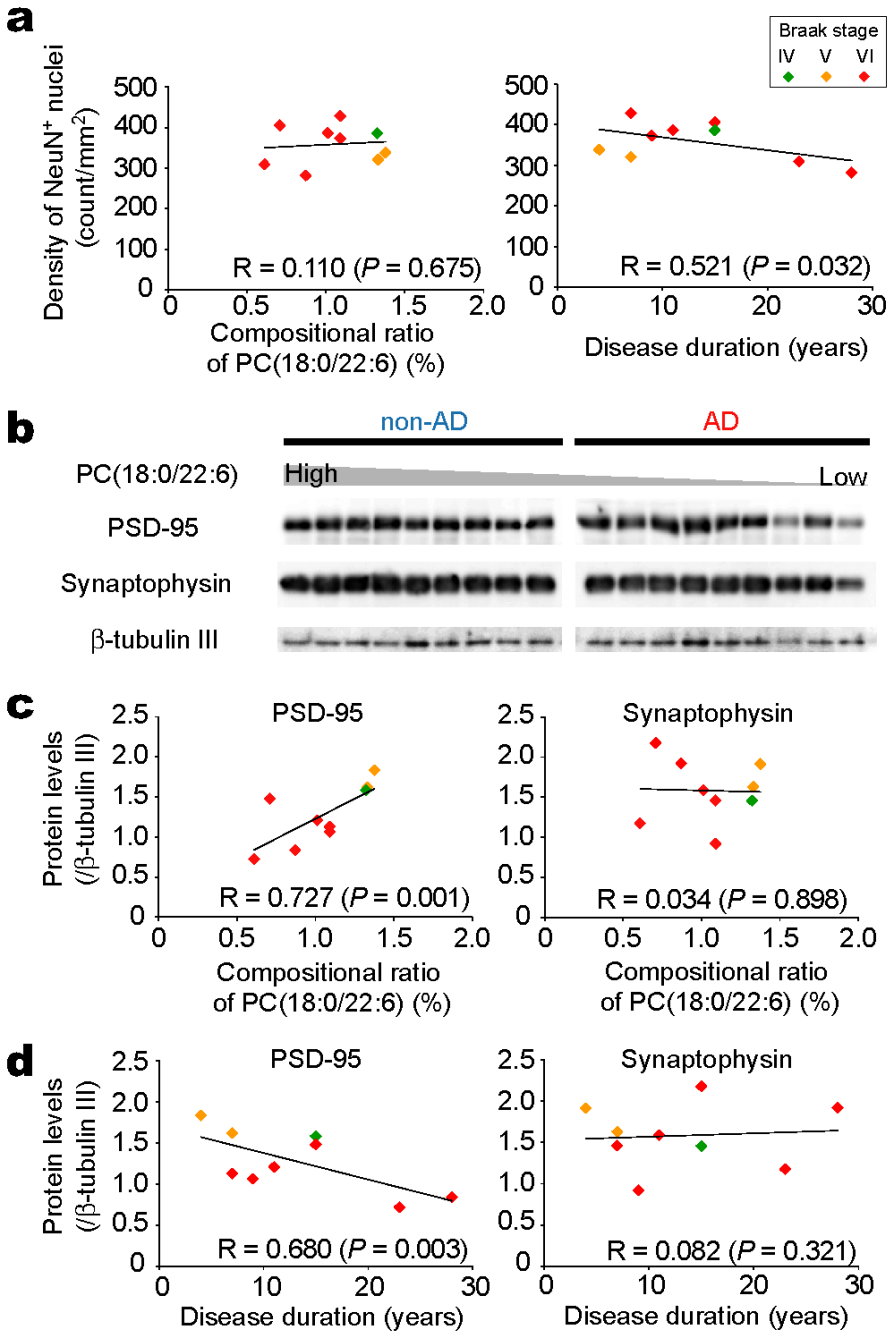
A decrease in the PC(18:0/22:6) concentration correlates with PSD-95 expression but not synaptophysin expression or neuron density in AD. (a) The NeuN-positive density in the gray matter plotted against the compositional ratio of PC(18:0/22:6) (left panel) and disease duration (right panel) in AD (n = 9). The colors of the markers indicate the Braak stages of each patient as shown in the top-right box. (b) Shown are the western blot data for PSD-95, synaptophysin, and the internal standard β-tubulin III in the temporal gray matter of non-AD and AD brains. The lanes were arranged in descending order of the compositional ratio of PC(18:0/22:6) in non-AD and AD brains, respectively. (c) The compositional ratio of PC(18:0/22:6) in the gray matter plotted against the protein levels of PSD-95 (left panel) and synaptophysin (right panel) in AD brains (n = 9). (d) Disease duration plotted against the protein levels of PSD-95 (left panel) and synaptophysin (right panel) in AD brains (n = 9). A Pearson's test was used to determine the correlations between the parameters.

We speculated that the observed depletion in PC(18:0/22:6) might be associated with neurodegenerative processes, such as neuronal loss and synaptic disruption, in gray matter regions of the AD brain. First, we examined the relationship between the decrease in PC(18:0/22:6) and neuron loss in the AD brain. Coronal temporal sections from non-AD and AD brains were immunostained with NeuN antibody, and the numbers of NeuN-positive nuclei were counted in the gray matter regions. The density of NeuN-positive nuclei in cortical layers II to VI in AD patients had a tendency to be less than those in non-AD patients. However, the density of NeuN-positive cells in the AD patients did not correlate with the compositional ratio of PC(18:0/22:6) (Fig. 5a, left panel), although the density of NeuN-positive cells negatively correlated with disease duration (Fig. 5a, right panel).

Second, we examined the relationship between PC(18:0/22:6) and synaptic disruption. We analyzed the expression levels of the presynaptic marker protein synaptophysin and the postsynaptic marker protein PSD-95 in the gray matter of non-AD and AD brains with immunoblotting (Fig. 5b). The protein levels for synaptophysin and PSD-95 were both decreased in the brains of AD patients. We then examined the correlations between the levels of synaptic proteins and the compositional ratio of PC(18:0/22:6) within the AD patients. A significant positive correlation was observed between PSD-95 and PC(18:0/22:6) (R = 0.727, *P* = 0.001), and no correlation was observed between synaptophysin and PC(18:0/22:6) (R = 0.034, *P* = 0.898) (Fig. 5c). The similar correlations were observed in the analyses within the late-onset AD patients; the compositional ratio of PC(18:0/22:6) correlated with the PSD-95 levels (R = 0.491, p = 0.045) but not with synaptophysin (R = 0.275, p = 0.285). We also examined the correlations between the synaptic protein levels and disease duration and found duration to be negatively correlated with PSD-95 (R = 0.680, *P* = 0.002) but not with synaptophysin (R = 0.082, *P* = 0.753) (Fig. 5d). In addition, we performed the correlational analyses between other synaptic proteins and PC(18:0/22:6) or disease duration, postsynaptic protein NR2B was strongly correlated with the PC(18:0/22:6) (R = 0.881, *P* = 0.001) and disease duration (R = 0.656, *P* = 0.004), and presynaptic protein Munc-18-1 was correlated with the PC(18:0/22:6) (R = 0.523, *P* = 0.031), but not with disease duration (R = 0.022, *P* = 0.932) (supplementary Fig. 1).

### The compositional ratio of PC(18:0/22:6) was not decreased in the brains of 10-month-old APP-tg mice

**Figure 6:**
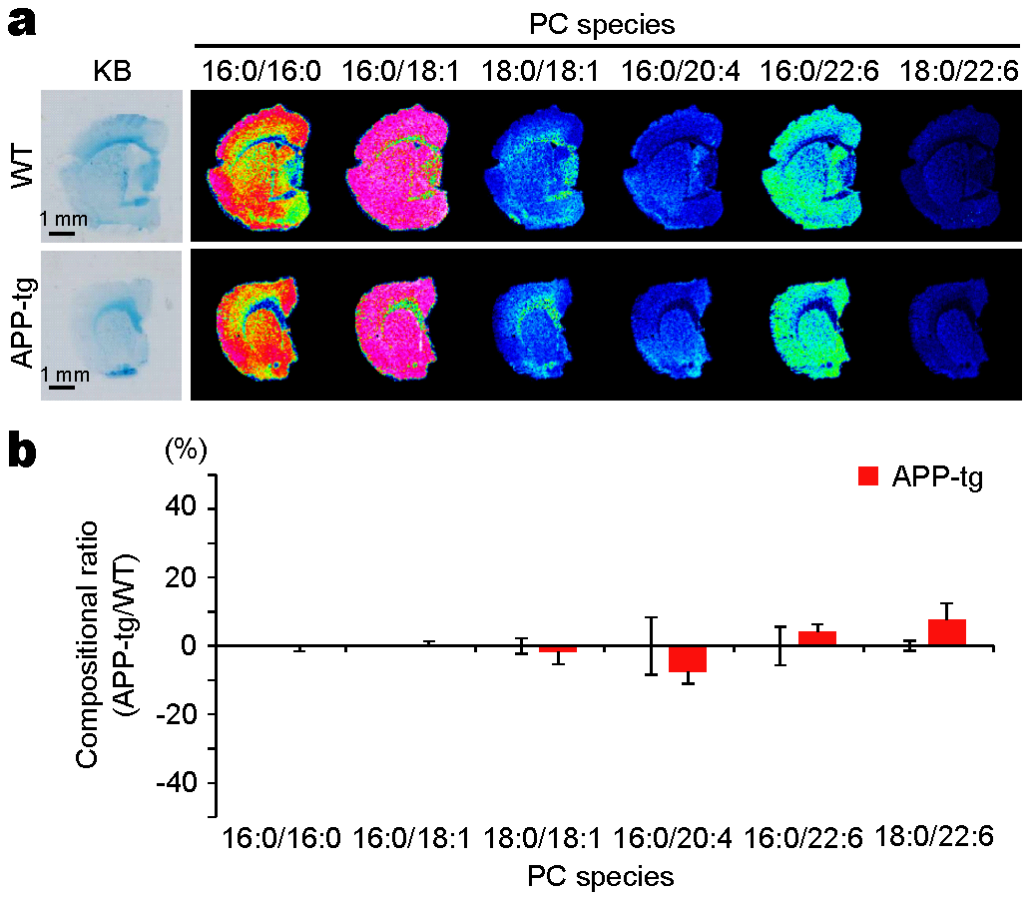
The compositional ratio of PC(18:0/22:6) did not decrease in the brain of an Amyloid precursor protein-transgenic (APP-tg) mouse. (a) Shown are the KB-stained sections and mass spectrometry (MS) images of the PC species in serial coronal sections of APP-tg (J20) and wild-type (WT) mice. The MS images are shown with 50 μm spatial resolution. The scale bars show 1 mm. (b) The graphs show the % change in the PC composition ratio between the APP-tg and WT mice in the gray matter. The data are shown as mean (SE). n = 3.

Finally, to see if PC(18:0/22:6) was reduced at the Aβ-accumulating stage before the postsynaptic disruptions, we performed a distributional analysis of PC in the brain of the human amyloid precursor protein (APP) transgenic (APP-tg) mouse (Fig. 6). Fig. 6a shows the distribution of the PC species in the coronal brain sections of 10-month-old APP-tg and wild-type (WT) mice. As was the case in the human brains, the distributions of the PCs in mice showed clearly different patterns between the gray and white matter, and PC(18:0/22:6) was distributed mainly in the gray matter. We did not find any significant differences in the contents of PC(18:0/22:6) in the gray matter of WT and APP-tg mice (Fig. 6b).

## Discussion

In this study, we identified six PC species in the human brains that we examined with MALDI-IMS (Fig. 1). These six PC species were accounted for by approximately 70 mol% of the total PC in the human brain when we used the quantitative results for PC that have been reported by Hermansson et al. as a reference^31^. Therefore, our results indicated that MALDI-IMS is a useful tool for revealing the distributional changes in major PC metabolism in the human brain.

The MALDI-IMS study of cerebral coronal sections showed that the differences in the distributions of the PC species were mainly between gray and white matter (Fig. 2b). This was consistent with the findings of previous studies that used extraction methods and that showed that the fatty acid composition of PCs in the human brain is clearly different between gray and white matter^32^. In a more detailed analysis with histograms of the intensity distributions in different brain regions, PC(16:0/22:6) and PC(18:0/22:6) were markedly decreased in the temporal gray matter region in the AD brain (Fig. 2c and d). These imaging results indicated that a decrease in DHA-PCs might be associated with neurodegeneration in the temporal gray matter region.

Previous studies on postmortem AD brains have reported a decrease in the total amount of PC^8^,^9^. In our quantitative analysis with LC-ESI MS/MS, the amounts of all of the PC species were decreased in both the gray and white matter of the temporal lobe in the brains of patients with AD (Fig. 3a, b). After converting the quantitative data into compositional ratios of the PC species, only the ratio of PC(18:0/22:6) was significantly decreased in the gray matter region (Fig. 3c). Therefore, all of the PC species were decreased in both the gray and white matter, and PC(18:0/22:6) was markedly decreased in the gray matter of AD brains.

The present study showed that the decrease in PC(18:0/22:6) did not correlate with the levels of Aβ deposition in the AD patient group (Fig. 4a). Interestingly, the PC(18:0/22:6) concentration was negatively correlated with disease duration in the AD patients (Fig. 4b), but this was independent of the age of the patients (Fig. 4c). In addition, the decrease in PC(18:0/22:6) was remarkable in the patients with Braak stage VI. AD is a progressive neurodegenerative disease, and the clinical symptoms develop over years. In a recently developed model of the disease stages of AD, Aβ deposition became abnormal early before the neurodegeneration and the clinical symptoms occurred^33^. Moreover, the deposition did not correlate with cognitive impairment in AD^18^. However, the loss of neurons and the synaptic disruption happened later than the Aβ deposition^34^, and they strongly correlated with the cognitive decline observed in AD^19^,^35^. Therefore, the progression of neurodegeneration is a more proximate pathological substrate of cognitive impairment in AD than Aβ deposition is^36^. Our result that the compositional ratio of PC(18:0/22:6) correlated with the duration of AD but not with Aβ deposition indicated that PC(18:0/22:6) was related to neurodegeneration in late-stage AD.

**Figure 7:**
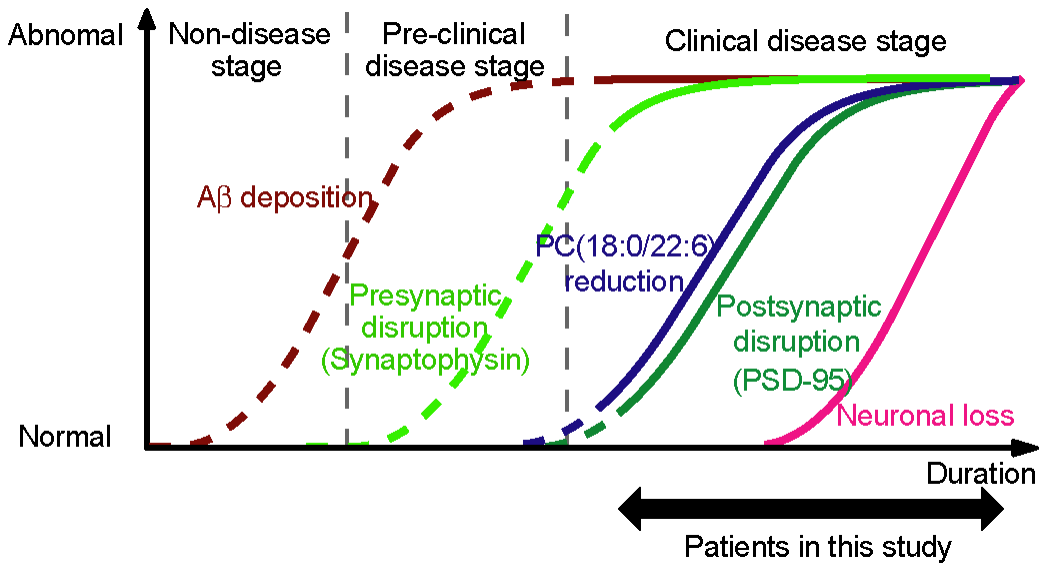
Hypothetical cascade in AD progression. A PC(18:0/22:6) reduction parallels postsynaptic disruption when plotted against disease duration, but it does not parallel Aβ deposition, neuronal loss, and presynaptic disruption. It is generally accepted that Aβ deposition becomes abnormal early and before the appearance of clinical symptom, and the abnormal brain morphology resulting from neuronal loss happens late relative to neuronal dysfunction^31^. Because the loss of synaptophysin was not correlated with the disease durations of the patients in this study, presynaptic disruption may occur earlier than postsynaptic disruption. The solid lines show the results of this study, and the broken lines show our speculation.

To examine the association with neurodegeneration in AD, we evaluated the possible relationship between the decrease in PC(18:0/22:6) and the loss of NeuN-positive cells (Fig. 5a). The density of NeuN-positive cells in AD brain did not correlate with a decrease in PC(18:0/22:6), but the density of the NeuN-positive cells negatively correlated with disease duration. These results indicated that the PC(18:0/22:6) reduction was not associated with neuronal loss. We next evaluated the relationship between the decrease in PC(18:0/22:6) and the loss of synaptic proteins (Fig. 5b). The postsynaptic protein PSD-95 was strongly correlated with PC(18:0/22:6) and disease duration (Fig. 5c and d). Similarly, the postsynaptic protein NR2B was strongly correlated with PC(18:0/22:6) and disease duration (Supplementary Fig. 1). However, the presynaptic protein synaptophysin did not correlate with PC(18:0/22:6) and disease duration, even though its levels were decreased in the AD brains. Although other presynaptic protein Munc-18-1 was correlated with PC(18:0/22:6), it did not correlate with disease duration (Supplementary Fig. 1). From these results, we hypothesized that the PC(18:0/22:6) was decreased at the clinical disease stage of AD and the reduction was more tightly associated with the postsynaptic disruptions than with the presynaptic disruptions (Fig. 7).

PSD-95 is a scaffold protein that is localized in postsynaptic terminals, and it has a critical role in postsynaptic function and plasticity^37^,^38^,^39^. A previous study with an AD mouse model (strain Tg2576) has reported that the loss of PSD-95 and other postsynaptic proteins is severe under a condition involving a dietary restriction of DHA, which has corresponding cognitive deficits^40^. Rodent studies have revealed that the brain's DHA content in phospholipids, such as PC, phosphatidylserine, and phosphatidylethanolamine, is decreased by dietary depletions of DHA^41^,^42^. However, a high DHA diet increases the DHA contents in the brain and increases the levels of PSD-95 and spine numbers^43^,^44^. Our results of the correlation of PC(18:0/22:6) and PSD-95 supported these previous studies and might indicate that PC(18:0/22:6) has an important role in the maintenance of postsynaptic function in humans.

Finally, we examined whether PC(18:0/22:6) was reduced at the early disease stage, which involves the accumulation of Aβ before postsynaptic disruption. We performed a MALDI-IMS analysis of brains from the 10-month-old AD model mouse J20 (Fig. 6) because J20 mice show Aβ deposition from the age of 8 months^45^,^46^, and spine loss, which reflects active postsynaptic disruption, is observed after 11 months of age^47^. As a result, the distribution and composition level of PC(18:0/22:6) were not significantly different between 10-month-old J20 and WT mice. This result suggested that the decrease in PC(18:0/22:6) did not occur at the early disease stage along with Aβ deposition. In other words, Aβ deposition was not a direct cause of the decreases in PC(18:0/22:6) in the brain. In a recent study, oxidative stress has been postulated to be the major effector of synaptic dysfunction in the AD brain^48^. DHA is easily oxidized by free radicals due to its high enrichment in double bonds. Indeed, biochemical studies have demonstrated the increased concentrations of reactive products from DHA peroxidation in diseased regions of the AD brain^49^. The cause of the compositional decrease of PC(18:0/22:6) in the AD brain might be oxidative stress, and this may contribute to accelerating the postsynaptic disruption in AD.

In this study, we found that PC(18:0/22:6) was characteristically depleted in the gray matter regions of AD brains. This decrease in the PC(18:0/22:6) concentration was significantly correlated with disease duration and the loss of PSD-95 protein but not with Aβ deposition or the loss of synaptophysin. These findings implied that, in the clinical stage of the disease, the depletion of PC(18:0/22:6) was linked to postsynaptic disruption, which occurs after the presynaptic disruption and before the neuronal loss.

## Methods

### Human brain

Postmortem brains from 10 AD patients and 10 non-AD subjects were provided from the Choju Medical Institute, Fukushimura Hospital, Toyohashi, Japan (Table 1). All of the AD patients were diagnosed with AD from the clinical symptoms, the Braak stage, and the Consortium to Establish a Registry for Alzheimer's Disease score at Fukushimura Hospital. The brains were removed at autopsy and cut midsagittally, and the left hemispheres were coronally divided into several regions. The divided tissue blocks were frozen on dry ice and stored at −80°C. The study was performed in accordance with the guidelines for pathological specimen handling, which was approved by the ethical committee of the Choju Medical Institute and Hamamatsu University School of Medicine.

### Animals

Human APP transgenic mice overexpressing the familial AD-linked mutations bearing both the Swedish (K670N/M671L) and Indiana (V717F) mutations (APP*Swe/Ind*)^45^, which were imported from The Jackson Laboratory (Bar Harbor, ME, USA), were obtained from the Laboratory of the School of Human Health Science, Kyoto University. They were maintained as heterozygotes, and male and female mice were housed separately. These mice were age- and sex-matched (1:1, male:female) and maintained on a standard diet (10% fat, 70% carbohydrate, and 20% protein, Oriental Yeast Co., Ltd., Tokyo, Japan). The brains were extracted and cut sagittally into left and right hemispheres. After removing the olfactory lobe and cerebellum, the right hemisphere was rapidly frozen in liquid nitrogen for the MALDI-IMS. All of the animal experiments were performed in compliance with the Guidelines for the Care and Use of Laboratory Animals of Kyoto University.

### Preparation of the tissue samples for MALDI-IMS

For MALDI-IMS, we used the large brain blocks that included the frontal, parietal, and temporal lobes from one AD patient (No. 1, Table 1) and one non-AD subject (No. 11, Table 1), which was used as a control. The human brain coronal tissue block was divided into four small blocks. The divided human tissue blocks and mouse brains were sectioned at −19°C with a cryostat (CM 1950; Leica Biosystems GmbH, Nussloch, Germany) to a thickness of 8 µm, as described previously^27^,^50^. The frozen sections were thaw-mounted onto a MALDI plate (Bruker Daltonics GmbH, Leipzig, Germany) or indium-tin-oxide-coated glass slides.

### Spray-coating with the matrix solution

A dihydroxybenzoic acid solution (50 mg/mL dihydroxybenzoic acid and 20 mM potassium acetate in methanol/water, 7:3, v/v) was used as the matrix solution. The matrix solution was sprayed over the tissue surface with a 0.2-mm caliber nozzle airbrush (Procon Boy FWA Platinum; Mr. Hobby, Tokyo, Japan). The distance between the nozzle tip and the tissue surface was maintained at 10 cm, and the spraying period was fixed at 5 min.

### The molecular characterization of PC and visualization of its distribution

The molecular characterization was performed with MS/MS and a MALDI linear quadrupole ion-trap mass-spectrometer (Thermo Fisher Scientific Inc., Waltham, MA, USA). The MS/MS analysis was performed directly on the brain sections. The acquisition was in the mid-mass-range mode (*m/z* 100–1,000), which is the positive-ion detection mode, with an ionization voltage of 30 V and a collision voltage of 35 V. The PC molecular species were identified from the neutral loss compositions, which were determined from the deltas of the precursor and product ions in the MS/MS spectra.

The IMS of the identified PC species was performed with a MALDI time-of-flight (TOF)/TOF-type instrument (Ultraflex II TOF/TOF; Bruker Daltonics GmbH) that was equipped with a 355-nm Nd:YAG laser. The data were acquired in the positive-ion reflector mode under an accelerating potential of 20 kV. Calibration was performed with an external calibration method. The signals of *m/z* = 700–1,000 were measured. Raster scans on the tissue surfaces were performed automatically with FlexControl and FlexImaging 2.0 software (Bruker Daltonics GmbH). The number of laser irradiations was 200 shots per spot. Image reconstruction was performed with FlexImaging 2.0 software.

### Sample preparation for LC-ESI MS/MS

For the LC-ESI MS/MS analysis, we analyzed the temporal lobes from the brains of nine AD patients (Nos. 2–10, Table 1) and nine non-AD subjects (Nos. 12–20, Table 1). The temporal lobes were sectioned at an 8-µm thickness and mounted on polymer-coated glass slides. Tissue sections with a 10-mm^2^ area were microdissected from the gray and white matter regions with a Leica LMD6500 system (Leica Biosystems GmbH). The microdissected tissues were collected into microtubes, and 10 μL of an internal standard solution [1 mg/mL PC(20:1/20:1) in methanol] was added. The total lipids were extracted by the Folch method^51^.

### LC-ESI MS/MS analysis

The LC-ESI MS/MS analysis was performed with a 4000Q-TRAP quadrupole linear ion-trap hybrid mass spectrometer (Applied Biosystems/MDS SCIEX, Concord, ON, Canada) that was connected to an ACQUITY Ultra Performance Liquid Chromatography system (Waters Corporation, Milford, MA, USA). A chromatographic method was developed with an ACQUITY UPLC BEH C18 column (2.1 mm i.d. × 50 mm, 1.7 µm; Waters Corporation) that was fitted with an identically packed guard column (2.1 mm i.d. × 5 mm, 1.7 µm; Waters Corporation). The column oven was maintained at 40°C. A gradient elution was used with a mobile phase A (acetonitrile:methanol:water = 19:19:2 v/v/v containing 0.1% formic acid and 0.028% ammonia) and a mobile phase B (isopropanol containing 0.1% formic acid and 0.028% ammonia). The protocol was as follows: flow rate = 0.4 mL/min: 0–10 min: 5% B, 10–15 min: 5% B→50% B, 15–20 min: 50% B, 20–25 min: 50% B→5% B. The MS/MS analysis was performed in the positive ESI mode with the following settings: ion spray voltage, 5,500 kV and temperature, 600°C. The detection of specific PC species was performed by multiple reaction monitoring. The [M + K]^+^ ions were selected in the first quadrupole (Q1) and collided with Ar in the second quadrupole (Q2) with a collision energy of 30 eV, and the product ions were detected at m/z 184 in the third quadrupole (Q3). The integrated signals of each monitored mass transition were corrected, and the signal of the internal standard PC(20:1/20:1) was used for quantification of each PC species.

### Immunohistochemistry

The temporal lobes from the brains of the AD and non-AD subjects (Nos. 2–10 and 12–20, Table 1) were sectioned at −19°C with a cryostat (CM 1950; Leica Biosystems GmbH) at a thickness of 8 µm and thaw-mounted onto glass slides. The tissue sections were fixed with 4% paraformaldehyde/phosphate-buffered saline for 10 min at room temperature. The slides were incubated for 10 min in 100% ethanol containing 3% hydrogen peroxide to inactivate endogenous peroxidases. Nonspecific sites were blocked by a 1-h exposure to 10% bovine serum albumin in Tris-buffered saline (TBS). Incubations with the mouse anti-human amyloid beta antibody (clone 82E1, 1:100) or the mouse anti-NeuN antibody (clone A60, 1:1,000) were performed in 0.1% Tween-20 in 3% bovine serum albumin TBS for 1 h at room temperature. The secondary antibody was goat anti-mouse IgG (Histofine Simple Stain^TM^ MULTI, Nichirei Biosciences Inc., Tokyo, Japan). After incubation with the secondary antibody and the ABC reagent (Thermo Fisher Scientific Inc., Rockford, IL, USA), sections were developed with metal-enhanced DAB kits (Thermo Fisher Scientific Inc.). Image analysis of the sections was performed with ImageJ software (NIH, Bethesda, MD, USA). The quantitative analysis of neuronal density (NeuN-positive cell count) was performed to produce an average in cortical layers II to VI.

### Sample preparation for immunoblotting

The temporal lobe gray matter tissue from non-AD and AD patients (Nos. 2–10 and 12–20, Table 1) were homogenized in 10 volumes of lysis buffer (TBS, 1 mM ethylenediaminetetraacetic acid, 2% sodium dodecyl sulfate, 0.5% deoxycholate) containing a cocktail of protease inhibitors (complete and ethylenediaminetetraacetic acid-free, Roche Diagnostics, Indianapolis, IN, USA). The samples were sonicated briefly and centrifuged at 100,000 × *g* for 20 min at 4°C. The supernatants were subjected to immunoblotting.

### Immunoblotting

Samples (20-µg protein) were electrophoresed on 10% acrylamide gels and transferred to polyvinylidene fluoride membranes (EMD Millipore Corporation, Billerica, MA). The membranes were blocked with TBS-T (0.1% Triton X-100 in TBS) containing 10% goat serum. The blot was rinsed with TBS-T and incubated at room temperature in TBS-T containing 2% goat serum and one of the following antibodies: anti-PSD-95 (clone K28/43, 1:100,000), anti-synaptophysin (clone SY38, 1:1,000), or anti-β-tubulin III (clone SDL.3D10, 1:1,000) for 90 min. Each membrane was then washed with TBS-T and incubated in TBS-T containing 2% goat serum and a 1:10,000 dilution of horseradish peroxidase-conjugated anti-mouse IgG for 60 min. Detection of the conjugated antibody was performed with ECL plus western blotting detection reagents (GE Healthcare Life Sciences, Freiburg, Germany). The fluorescence intensity of the immunoblotted proteins was quantified with ImageJ software and calibrated by using the β-tubulin III signal as an internal standard.

### Statistical analysis

The data are presented as mean ± standard error. The statistical comparison of PC species levels between the AD and non-AD brains was performed with a Student's *t*-test. Differences were considered significant with p values less than 0.05. Pearson's test was used for the correlational analyses between the compositional ratio of PC(18:0/22:6) and Aβ deposition, disease duration, age at death, neuron density, and synaptic protein levels.

## Supplementary Material

Supplementary Information Supplementary Figure 1

## Figures and Tables

**Table 1:** Demographic data for patients with Alzheimer's disease (AD) and non-AD subjects

No.	Diagnosis	Gender	Age at death (years)	Duration of disease (years)	Braak stage	CERAD score	Postmortem interval (hours)	Clinical diagnosis
1	AD	male	80	10	VI	C	12	Alzheimer's disease, Dementia with Lewy bodies
2		female	83	9	VI	C	not recorded	Alzheimer's disease, Dementia with Lewy bodies
3		female	91	15	VI	C	20	Alzheimer's disease, Vascular dementia
4		female	93	7	VI	C	3	Alzheimer's disease, Cerebral amyloid angiopathy
5		female	91	4	V	C	18	Alzheimer's disease
6		male	81	11	VI	C	8	Alzheimer's disease, Acute respiratory failure
7		male	91	7	V	C	5	Alzheimer's disease
8		male	90	15	IV	C	not recorded	Alzheimer's disease, Progressive supranuclear palsy
9		female	86	28	VI	C	2	Alzheimer's disease, Cerebral amyloid angiopathy
10		female	75	23	VI	C	4	Alzheimer's disease
11	non-AD	male	88	0	I	0	20	hypoglycemia, pneumonia
12		male	79	0	I	B	13	multiple cerebral infarction
13		male	80	0	I	0	not recorded	multiple cerebral infarction
14		male	87	0	II	0	10	Cerebral infarction
15		female	82	0	I	0	34	Cerebral infarction
16		female	83	0	I	0	28	Phigiological aging
17		male	80	0	I	0	2	Phigiological aging
18		female	95	0	I	A	4	multiple cerebral infarction
19		female	86	0	II	A	4	multiple cerebral infarction
20		male	88	0	II	B	8	Cerebral infarction
