# Supplementary material for: DHA-PC and PSD-95 decrease after loss of synaptophysin and before neuronal loss in patients with Alzheimer's disease
Source: Sci Rep. 2014 Nov 20;4:7130. doi: 10.1038/srep07130 (PMC5382699; doi:10.1038/srep07130)
Supplement: Supplementary Information — Supplementary Figure 1 [file srep07130-s1.pdf]

Supplementary Information

DHA-PC and PSD95 decrease after loss of synaptophysin and before neuronal loss in patients with Alzheimer's disease

Dai Yuki[1, 2], Yuki Sugiura[1, 3], Nobuhiro Zaima[1, 4], Hiroyasu Akatsu[5, 6], Shiro Takei[7], Ikuko Yao[7], Masato Maesako[8], Ayae Kinoshita[8], Takayuki Yamamoto[5], Ryo Kon[2], Keikichi Sugiyama[2, 9], Mitsutoshi Setou[1]*

[1]Department of Molecular Anatomy, Hamamatsu University School of Medicine, 1-20-1 Handayama, Higashi-ku, Hamamatsu, Shizuoka 431-3192, Japan

[2]Research and Development Headquarters, Lion Corporation, 7-2-1 Hirai, Edogawa-ku, Tokyo 132-0035, Japan

[3]JST Precursory Research for Embryonic Science Technology (PREST) Project, 160-8582 Tokyo, Japan

[4]Department of Applied Biological Chemistry, Kinki University, 3327-204 Naka-machi, Nara 631-8505, Japan

[5]Choju Medical Institute, Fukushimura Hospital, 19-14 Yamanaka, Noyori-cho, Toyohashi, Aichi 441-8124, Japan

[6]Department of Medicine for Aging in Place and Community-Based Medical Education, Nagoya City University Graduate School of Medical Sciences, Nagoya, Aichi 467-8601, Japan

[7]Department of Optical Imaging, Hamamatsu University School of Medicine, 1-20-1 Handayama, Higashi-ku, Hamamatsu, Shizuoka 431-3192, Japan

[8]School of Human Health Sciences, Kyoto University Graduate School of Medicine, 53 Shogoin kawahara-cho, Sakyo-ku, Kyoto 606-8507, Japan

[9]Ritsumeikan Global Innovation Research Organization, Ritsumeikan University, 1-1-1 Nojihigashi, Kusatsu, Shiga 525-8577, Japan

*Corresponding author: Department of Molecular Anatomy, Hamamatsu University School of Medicine, 1-20-1 Handayama, Higashi-ku, Hamamatsu, Shizuoka 431-3192, Japan. Tel: +81-53-435-2292; fax: +81-53-435-2292. E-mail address: setou@hama-med.ac.jp (M. Setou).

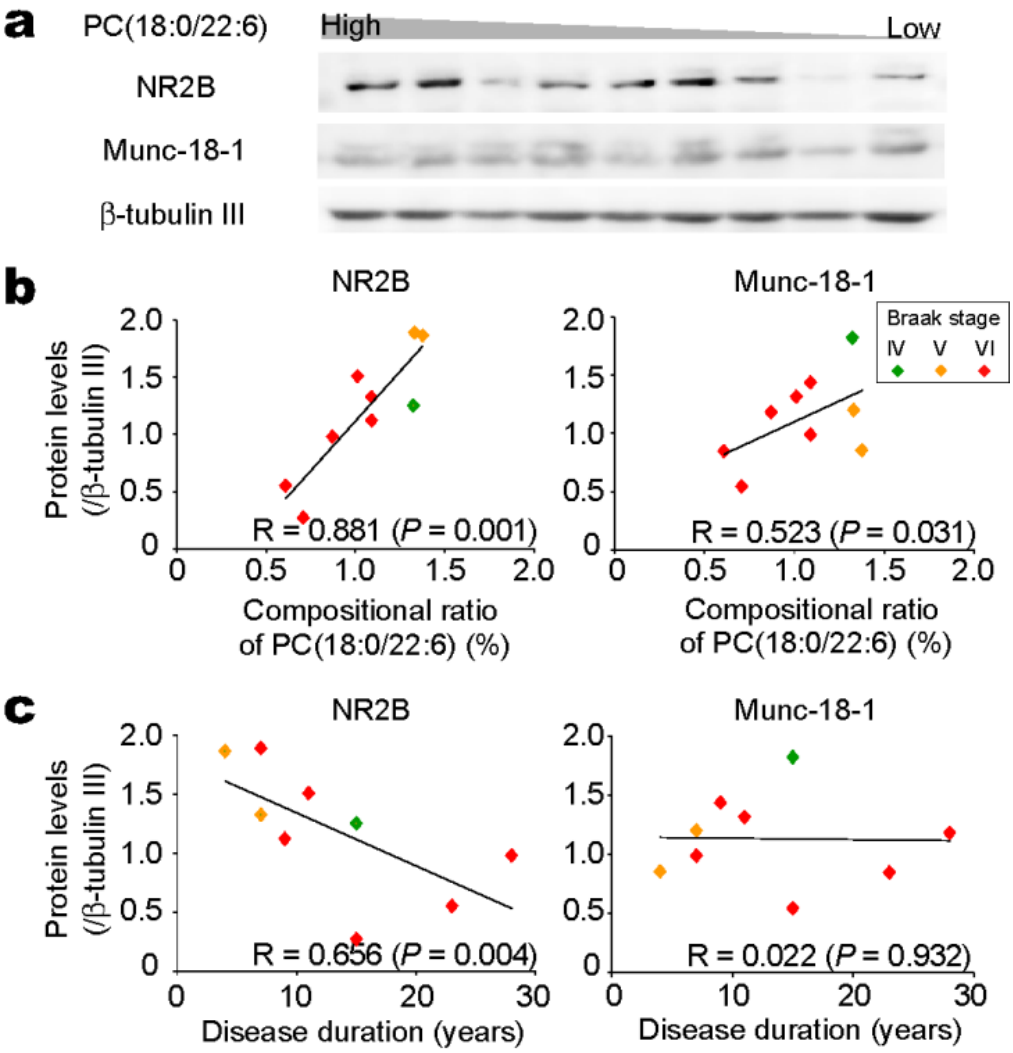

Figure S1 | A decrease in the PC(18:0/22:6) concentration correlates with NR2B and Munc-18-1 expression in AD. (**a**) Shown are the western blot data for NR2B, Munc-18-1, and the internal standard β-tubulin III in the temporal gray matter of AD brains. The lanes were arranged in descending order of the compositional ratio of PC(18:0/22:6) in AD brains. (**b**) The compositional ratio of PC(18:0/22:6) in the gray matter plotted against the protein levels of NR2B (left panel) and Munc-18-1 (right panel) in AD brains (n = 9). The colors of the markers indicate the Braak stages of each patient as shown in the right box. (**c**) Disease duration plotted against the protein levels of NR2B (left panel) and Munc-18-1 (right panel) in AD brains (n = 9). A Pearson's test was used to determine the correlations between the parameters.
